# Unveiling the Impact of ApoF Deficiency on Liver and Lipid Metabolism: Insights from Transcriptome-Wide m6A Methylome Analysis in Mice

**DOI:** 10.3390/genes15030347

**Published:** 2024-03-09

**Authors:** Xuebin Shen, Mengting Chen, Jian Zhang, Yifan Lin, Xinyue Gao, Jionghong Tu, Kunqi Chen, An Zhu, Shanghua Xu

**Affiliations:** 1 Department of Cardiology, Affiliated Nanping First Hospital, Fujian Medical University, Nanping 353000, China; sxbzsh@fjmu.edu.cn (X.S.); linyifan12@fjmu.edu.cn (Y.L.); gaoxinyue@fjmu.edu.cn (X.G.); 2 Key Laboratory of Ministry of Education for Gastrointestinal Cancer, School of Basic Medical Sciences, Fujian Medical University, Fuzhou 350108, China; chenmengting@fjmu.edu.cn (M.C.); 3220119035@stu.fjmu.edu.cn (J.T.); kunqi.chen@fjmu.edu.cn (K.C.)

**Keywords:** RNA modification, m6A, MeRIP-seq, apolipoprotein, lipid metabolism

## Abstract

Lipid metabolism participates in various physiological processes and has been shown to be connected to the development and progression of multiple diseases, especially metabolic hepatopathy. Apolipoproteins (Apos) act as vectors that combine with lipids, such as cholesterol and triglycerides (TGs). Despite being involved in lipid transportation and metabolism, the critical role of Apos in the maintenance of lipid metabolism has still not been fully revealed. This study sought to clarify variations related to m6A methylome in *ApoF* gene knockout mice with disordered lipid metabolism based on the bioinformatics method of transcriptome-wide m6A methylome epitranscriptomics. High-throughput methylated RNA immunoprecipitation sequencing (MeRIP-seq) was conducted in both wild-type (WT) and *ApoF* knockout (KO) mice. As a result, the liver histopathology presented vacuolization and steatosis, and the serum biochemical assays reported abnormal lipid content in KO mice. The m6A-modified mRNAs were conformed consensus sequenced in eukaryotes, and the distribution was enriched within the coding sequences and 3′ non-coding regions. In KO mice, the functional annotation terms of the differentially expressed genes (DEGs) included cholesterol, steroid and lipid metabolism, and lipid storage. In the differentially m6A-methylated mRNAs, the functional annotation terms included cholesterol, TG, and long-chain fatty acid metabolic processes; lipid transport; and liver development. The overlapping DEGs and differential m6A-modified mRNAs were also enriched in terms of lipid metabolism disorder. In conclusion, transcriptome-wide MeRIP sequencing in *ApoF* KO mice demonstrated the role of this crucial apolipoprotein in liver health and lipid metabolism.

## 1. Introduction

Lipid metabolism is the biochemical reaction of lipids in various metabolic enzymatic reactions and is involved in multiple physiological processes, including digestion, absorption, synthesis, metabolism, and signal transduction [1]. Lipid metabolism plays a crucial role in maintaining cellular homeostasis, which includes processes like lipid uptake, synthesis, and hydrolysis. In organisms, cholesterol and triglycerides (TGs) are the primary lipids that are insoluble in water and need to bind with circulating plasma apolipoproteins (Apos) to form lipoproteins, thus enabling the transportation and metabolism of lipids [2]. The liver is the central organ responsible for lipid metabolism due to its involvement in the synthesis of lipoproteins for lipid transportation as well as fat synthesis and breakdown [3].

ApoB, A-I, and E contribute positively to the structural stability of lipoproteins [4]. These can be classified into various subtypes, with ApoA1 being an essential component of high-density lipoprotein cholesterol (HDL-C) and playing a protective role against atherosclerosis by participating in cholesterol reverse transport [5,6]. On the other hand, ApoB is found in chylomicrons and low-density lipoprotein cholesterol (LDL-C). Cholesteryl ester transfer protein (CETP) is responsible for facilitating the transfer of cholesteryl esters (CEs) from HDLs and LDLs to very low-density lipoproteins (VLDLs), exerting significant influence on the composition and metabolism of lipoproteins. However, the activity of CETP can be inhibited by ApoF, which prevents CETP from binding to the phospholipids on the surface of lipoproteins, thereby promoting cholesterol reverse transport [7]. ApoF is primarily expressed in the liver and predominantly located in LDL and HDL [8]. In C57BL/6 mice, hepatic *ApoF* mRNA expression levels increased upon treatment with farnesioid X receptor (FXR) agonists. This suggests that the 5′ flanking region of the *ApoF* gene promoter contains the FXR response element-binding site ER1 (−2904 to −2892 bp) [9]. Multiple putative binding sites for transcription factors E26 transformation-specific (ETS) and CCAAT/enhancer-binding protein (C/EBP) were identified in the −198 nt to −2 nt region of the *ApoF* promoter. ETS-1 can interact with C/EBPα and synergistically activate *ApoF* transcription [10]. Epidemiological data from the US have demonstrated a positive correlation between ApoF levels in human plasma and HDL levels as well as a negative correlation with TG levels. Therefore, ApoF is considered a protective factor against hyperlipidemia [11]. Abnormal expression levels of ApoF have been reported in various diseases and animal models. In patients with coronary heart disease, a significant decrease in ApoF levels in HDLs has been observed [12]. In male golden Syrian hamsters fed a high-cholesterol diet, hepatic *ApoF* mRNA expression was downregulated [13]. Although the involvement of ApoF in lipid metabolism has been confirmed, the exact molecular mechanism is still poorly understood.

Epitranscriptomics, a term used to describe the regulation of gene expression through RNA modifications, has been implicated in lipid metabolism. To date, more than 170 modifications are present in RNA epitranscriptomics, including N6-methyladenosine (m6A) [14,15,16], which is considered the primary internal modification found in mRNA within eukaryotic cells [17]. RNA m6A modification is dynamically reversible and is regulated by m6A methyltransferases (writers), demethylases (erasers), and binding proteins (readers) [18]. The regulation of lipid metabolism by RNA m6A methylation relies on the modulation of m6A levels and recognition by different regulatory enzymes, but the precise mechanisms underlying this effect have not been completely elucidated. The first identified RNA m6A demethylase fat mass and obesity associated (FTO) plays a pivotal role in lipid metabolism. FTO induces lipid accumulation by reducing the m6A methylation levels of various lipogenic genes like *SREBP1* [19]. The distribution of FTO in various tissues includes adult and fetal livers, adipose tissue, islets, skeletal muscle, and macrophages [20]. Knockout of *FTO* leads to an upregulation of interleukin-6 expression in adipose tissue, thereby facilitating the upregulation of lipolysis genes and ameliorating hepatic steatosis in mice livers [21]. FTO can decrease mRNA stability and suppress the expression of *ApoE* and the glucose metabolism pathway via IGF2BP2-mediated m6A modification, thereby impacting the progression of papillary thyroid cancer [22]. Li et al. [23] demonstrated that levels of m6A were consistently upregulated in mice models, with liver metabolic disorders induced by a high-fat diet. The m6A reader YTH N6-methyladenosine RNA-binding protein F2 (YTHDF2) modulates lipid metabolism by facilitating the degradation of mRNA transcripts that target the recognition of m6A modifications [24]. The m6A regulators are involved in lipid metabolism regulation, with ApoF playing a critical role. Following knockout of the *ApoF* gene, it is crucial to explore whether there are any changes in the transcriptome-wide m6A methylome profile and the regulatory mechanism of lipid metabolism.

In this study, a C57BL/6 mouse model with *ApoF* gene knockout was constructed. The transcriptome-wide m6A methylome profile was then analyzed using next-generation high-throughput sequencing. The differentially expressed mRNA and m6A-modified genes were enriched to investigate the role of ApoF in liver lipid metabolism.

## 2. Materials and Methods

### 2.1. Lipid Metabolism Disorder Mice with ApoF Gene Knockout

The model of *ApoF* gene knockout in male C57BL/6 mice was constructed by GemPharmatech (Nanjing, China). The *ApoF* gene has two transcripts, and exon1-exon2 of the *ApoF*-202 (ENSMUST00000238970) transcript is regarded as the targeted knockout region according to the structure of the *ApoF* gene. Eliminating this region would render the protein dysfunctional. Briefly, clustered regularly interspaced short palindromic repeats (CRISPR)/CRISPR-associated (Cas9) technology was used to modify the *ApoF* gene. The sgRNA1 sequence (5′ ⟶ 3′) of 5S1 was CAGCAAACTCGCTGACCATG, bound with a protospacer adjacent motif (PAM) sequence of TGG, and the 3S1 sequence of sgRNA2 was CAGGGTGCGTGAAGGCACCT, bound with a PAM sequence of AGG. In vitro transcription was employed to generate the sgRNAs. The fertilized eggs of C57BL/6 mice microinjected with both Cas9 and sgRNA were transplanted to yield F0 mice. The available F1-generation mouse model was acquired by breeding positive F0-generation C57BL/6 mice. The male mice were fed in an SPF house and acclimated to the environment for 1 week before the experiments. The ambient temperature was controlled at 20 °C while following a 12-h light and dark cycle, and a commercial diet was provided. The study included 12 mice in total, distributed across four groups, 8-week-old WT group, 8-week-old KO group, 28-week-old WT group, and 28-week-old KO group, with three mice in each group. Approval for this research was obtained from the Medical Ethics Committee of Nanping First Hospital (No. NPSY202102002) in year 2021. RNA isolated from tails of the 3-week-old juvenile mice were used to identify genotypes with the primers of *ApoF* F1 (TCTCTGTATAGCCTTGTCTGCCCA), R1 (CAAGATAGCGAACAGCGGAATG), F2 (CTCTAAGGAATGCTCTGGAGGCA), and R2 (TGCCTACTGCTGGTAGTAGGTCAAC). The resulting products were then subjected to sequence analysis using a 3730xl DNA Analyzer (Thermo Fisher, Waltham, MA, USA).

Tails of mice underwent lysis and amplification using TransDirect Animal Tissue PCR Kit (Transgen Biotech, Beijing, China). Genotype identification of the amplified DNA samples was then conducted using DNA gel electrophoresis in a 1% agarose gel at 140 V.

### 2.2. Histopathological Examination of Liver Tissue

To investigate the relationship between liver injury and ApoF deficiency, liver histopathological observation was performed using hematoxylin and eosin (H&E) staining techniques. Hematoxylin is a basic dye used for staining the nucleus blue, while eosin is an acidic dye used for staining the cytoplasm red [25]. For histopathological evaluation, liver tissue was collected and fixed in 4% paraformaldehyde. Briefly, the tissue was rinsed with a phosphate-buffered saline (PBS) solution, dehydrated in an ethanol gradient, and then embedded in paraffin. The 5 µm liver tissue was cut into sections and heat dried, followed by dehydration, dewaxing, and H&E staining (Meilune, Dalian, China). The histomorphological changes in mouse livers were observed under a microscope (Nikon, Tokyo, Japan).

### 2.3. CT Image Acquisition

The 28-week-old mice were positioned in the supine position after the onset of anesthesia via isoflurane inhalation. Spiral CT (GE Healthcare, Chicago, IL, USA) was used for the nonenhanced scan to get the energy spectrum curve. The scanning range extended from the dome of the diaphragm to the lower edge of the liver. The gemstone spectral imaging scanning mode was selected, and the parameters were as follows: the tube voltage was switched every 0.5 ms, and the value was set to 80 kVp or 140 kVp; the tube current was 190 mA; the rotation time was 0.5 s/turn; and slice and spacing thicknesses were 0.625 mm. Subsequently, the images acquired were imported into the workstation. The region of interest (ROI) was above the first hepatic portal of the right lobe of the liver, with an approximate area of 4.85 mm². Three slices were selected to average the ROI for each mouse, and then the energy spectrum curve was generated.

### 2.4. Measurement of Serum Transaminase Enzymes and Blood Lipids

In order to evaluate the influence of ApoF on hepatic function, the activities of serum biomarkers alanine aminotransferase (ALT) and aspartate transaminase (AST) were analyzed. Additionally, to quantitatively confirm the effect of ApoF on lipid metabolism, the contents of TG and HDL-C in serum were measured. Briefly, approximately 1 mL of peripheral blood was collected from each mouse, aged 8 weeks and 28 weeks, and transferred into tubes. The tubes were then placed on ice and allowed to settle for 30 min. The serum was separated from the blood via centrifugation at 3000× *g* for 10 min to measure the levels of serum ALT, AST, TG, and HDL-C using enzymatic colorimetric methods in an automatic biochemical analyzer AU5821 (Beckman Coulter, Brea, CA, USA).

### 2.5. RNA Isolation and Extraction

Approximately 100 mg of liver tissue from mice aged 8 weeks and 28 weeks was harvested and placed in a TRIzol reagent (Invitrogen, Carlsbad, CA, USA) to homogenize. Total RNA was extracted using chloroform, isopropanol, and ethanol. The RNA concentration was detected by Qubit3.0 (Thermo Fisher), and RNA integrity was determined using a fragment analyzer 5300 (Agilent, Santa Clara, CA, USA).

### 2.6. High-Throughput m6A MeRIP-seq and mRNA-seq

The process of enriching RNAs with m6A methylation modifications and high-throughput MeRIP-seq was performed by Seqhealth (Wuhan, China). In brief, polyadenylated RNA was enriched in 10 μg total RNA by beads (Vazyme, Nanjing, China). Then, the mRNA was incubated in the medium until the mRNA was disrupted into 100 to 200 nucleotides in length, and the m6A antibody (Synaptic Systems, Goettingen, Germany) was used for m6A immunoprecipitation. A portion of the original fragments of mRNAs was retained as the input. The input mRNAs and the m6A antibody-enriched mRNAs were used to construct RNA libraries prepared by the KC-DigitalTM Stranded mRNA Library Prep Kit for Illumina (Seqhealth, Wuhan, China). Subsequently, library products with 200–500 bps were enriched, quantified, and ultimately processed on a DNBSEQ-T7 sequencer (MGI Technology Co., Ltd., Shenzhen, China). Three independent samples in each group were used for sequencing. The sequence data were uploaded to the public database GEO.

### 2.7. RT-qPCR

1000 ng RNA was reverse transcribed into cDNA using the Evo M-MLV RT Kit with gDNA Clean for qPCR II (Accurate Biotechnology, Changsha, China). Real-time quantitative PCR was performed using the SYBR Green Premix Pro Taq HS qPCR Kit (Accurate Biotechnology). To amplify the target genes, the following conditions were used: initial heat activation at 95 °C for a duration of 30 s, 40 cycles of denaturation at 95 °C for 5 s, annealing at 60 °C for 30 s, and a final step of 95 °C for 5 s. The 2^−ΔΔCT^ method was employed to determine the relative expression of the target gene, with *GAPDH* serving as the internal reference gene. Table 1 exhibits the PCR primer sequences utilized in this investigation, which were designed using Primer-BLAST (http://blast.ncbi.nlm.nih.gov/, accessed on 23 November 2022) and synthesized by Sangon Biotech (Shanghai, China). In the qPCR reactions, the primers were used at a final concentration of 10 μM and a volume of 0.8 μL.

### 2.8. Bioinformatic Analysis

The raw MeRIP-seq data were aligned to genome reference sequences (mm10/GRCm38, download from Genome Browser, accessed on 24 December 2022) by Hisat2 with default parameters [26]. The aligned reads were used for m6A-modification peak calling, whereby significant methylation was identified using exomepeak2 (Suzhou, China) [27,28], and m6A peak calling was visualized using IGV 2.16.2 software. The distribution pattern of epitranscriptome profiles was examined using MetaTX (Suzhou, China) [29]. The R version of mouse genome reference “BSgenome.Mmusculus.UCSC.mm10” was used for peak calling and pattern visualization; otherwise, parameters with default settings were applied. STREME [30] was used to determine if the m6A peaks contained the m6A motif consensus sequences (minimum width: 5; maximum width: 10; *p* value < 0.05). For mRNA-seq, the mRNA expression level was analyzed using StringTie (with parameters -eB -p 20, version 1.3.6, Baltimore, MD, USA) [31], and differentially expressed mRNAs were calculated using DEseq [32]. The m6A regulator substrates were obtained from starBase v2.0 (Guangzhou, China) [33]. The enrichment analysis of Gene Ontology (GO) and the Kyoto Encyclopedia of Genes and Genomes (KEGG) for DEGs and differentially methylated mRNAs were applied using DAVID (Frederick, MD, USA) [34] and KOBAS (Beijing, China) [35], respectively. GO enrichment analysis involves comparing a list of genes of interest with a background set of genes to discern overrepresented biological processes, molecular functions, and cellular components. Conversely, KEGG pathway analysis focuses on identifying significantly enriched biological pathways within a given list of genes or proteins. The substrates of the m6A regulators were predicted by POSTAR3 (Beijing, China) [36].

### 2.9. Western Blotting

RIPA lysate containing phosphatase and proteinase inhibitors (Beyotime, Shanghai, China) was configurated to extract protein from the livers of mice aged 8 weeks and 28 weeks. Protein concentrations were measured using a standard curve constructed with bovine serum albumin (BSA) standard protein in a BCA kit (Vazyme, Nanjing, China). Then, the proteins were denatured at 100 °C for 10 min. Equal amounts of protein were separated via sodium dodecyl sulfate-polyacrylamide gel electrophoresis and subsequently transferred to PVDF membranes at 160 mA for 120 min. Membranes were then blocked with 5% skimmed milk for 90 min at room temperature. Then, the sample were rinsed three times with the Tris-saline-Tween 20 (TBST) buffer for 10 min. The target proteins on the membranes were subjected to overnight incubation with primary antibodies (Proteintech and ABclonal, Wuhan, China) at 4 °C. The antibody dilutions of ApoF, IGF2BP3, and GAPDH were 1:1000, 1:6000, and 1:10,000, respectively. Then, secondary antibodies were added and incubated for 1 h. The proteins on the membrane were exposed to ECL ultra-high-sensitivity luminescent liquid and imaged using an Amersham imager 680 system (Cytiva, Marlborough, MA, USA).

### 2.10. Statistical Analysis

Statistical analysis was performed using IBM SPSS 26.0 software (IBM, New York, NY, USA). Data are presented as the mean ± standard deviation (SD). Group differences were examined through one-way analysis of variance (ANOVA). The test of homogeneity of variances evaluated whether the variance was consistent across the data. If the significance level of the test was *p* > 0.05, the assumption of homogeneity of variances was not violated, and the least-significant difference test was used for post-hoc tests; however, if *p* < 0.05, Dunnett’s T3 test was employed for post-hoc tests. Statistical significance was established if the *p* value was below 0.05.

## 3. Results

### 3.1. Genotype Identification

**Figure 1:**
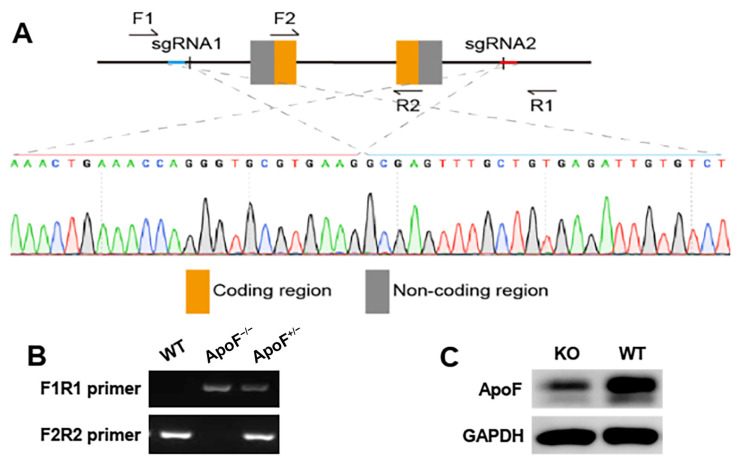
(**A**) The *ApoF*^−/−^ mice were sequenced. (**B**) The genotype identification of WT, *ApoF*^−/−^, and *ApoF*^+/−^ mice. (**C**) The ApoF protein expression decreased in *ApoF*^+/−^ mice.

Firstly, the mouse gene knockout model was verified (the *ApoF* gene was sequenced and is shown in Figure 1A), and exons 1 and 2 were knocked out. The DNA was extracted from the tails of 3-week-old mice. Following PCR amplification, the genotypes were determined using agarose gel electrophoresis, and the results are presented in Figure 1B. The ApoF protein expression was detected in 8-week-old mice (Figure 1C), which revealed a noticeable decrease in ApoF in the heterozygotes compared to the wild-type mice. These observations indicate the successful establishment of the mouse model.

### 3.2. Histopathological Observation and Detection of Enzyme Activity and Dyslipidemia in ApoF Knockout Mice

**Figure 2:**
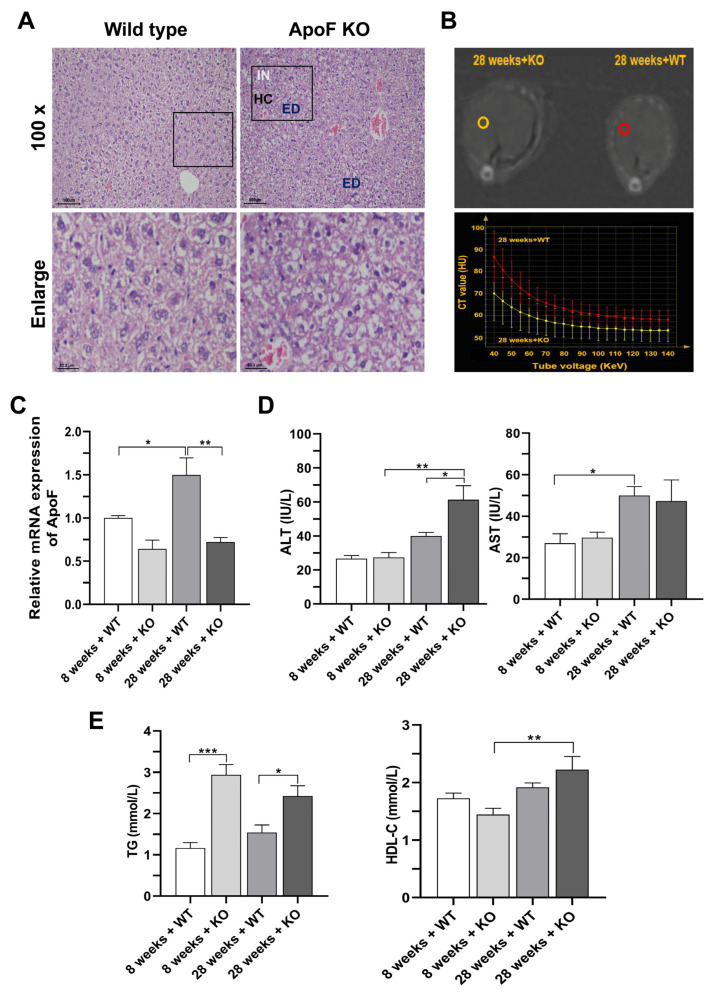
(**A**) Histopathological examination of the hematoxylin and eosin staining of the liver tissues of the 28-week-old mice. (**B**) ROI and energy spectrum curves of 28-week-old mice livers. (**C**) The mRNA expression levels of *ApoF* in mice livers. The effects of *ApoF* gene knockout on the (**D**) enzyme activities and (**E**) lipid profiles in serum. IN: inflammation infiltration; ED: edema; HC: disturbed hepatic cord. *n* = 3, * *p* < 0.05, ** *p* < 0.01, *** *p* < 0.001.

To investigate whether *ApoF* knockout could induce liver injury, we examined the pathological sections and energy spectrum curve of the liver and levels of ALT and AST in serum. As shown in Figure 2A, the liver tissue of wild-type mice was intact, and the hepatic sinusoids were clear and radially arranged around the central vein. There were no apparent signs of cell degeneration or necrosis. However, in the *ApoF* knockout mice, the overall structure of the liver was abnormal, the hepatic cord was disturbed, the hepatocytes were obviously edematous and enlarged, and the cytoplasm was loose and hyaline, along with a large number of infiltrating inflammatory cells. In the same ROI, energy spectrum curves representing fat content in 28-week-old KO mice were flatter than those in 28-week-old WT mice (Figure 2B). This finding suggests that the liver damage observed could be attributed to fat deposition resulting from the knockout of the *ApoF* gene.

*ApoF* mRNA expression was measured using RT-qPCR, as depicted in Figure 2C. According to the findings, there was an approximately 36% decrease in *ApoF* expression in the 8-week-old KO group when compared to the WT group. In addition, the mRNA expression of *ApoF* in the knockout mice was further decreased to about 50% of that in the 28-week-old wild-type mice (*p* < 0.01). In Figure 2D, the ALT activity in the 8-week-old WT, 8-week-old KO, 28-week-old WT, and 28-week-old KO groups was 26.67 ± 3.21, 27.3 ± 5.03, 40.00 ± 3.61, and 61.3 ± 14.29 IU/L, respectively. Compared to the 28-week-old WT group, the ALT activity in the 28-week-old KO group was higher (*p* < 0.05). Notably, with the prolonged duration of *ApoF* knockout, the ALT activities in the 28-week-old KO group were higher compared to the 8-week-old KO group (*p* < 0.01). The activities of AST were 27.00 ± 7.94, 29.67 ± 4.51, 50.00 ± 7.55, and 47.33 ± 17.61 IU/L in the 8-week-old WT, 8-week-old KO, 28-week-old WT, and 28-week-old KO groups, respectively. Abnormal liver structure and elevated serum transaminase enzyme levels suggested that the *ApoF* knockout caused liver injury. Moreover, in order to investigate whether the *ApoF* gene leads to dyslipidemia, serum TG and HDL-C contents were measured. As shown in Figure 2E, TG content of the 8-week-old WT group was lower than that of the 8-week-old KO group (*p* < 0.001). Correspondingly, the TG content of the 28-week-old KO group was significantly higher compared to the 28-week-old WT group (*p* < 0.05). The HDL-C levels showed significant differences between the 8th week and 28th week in the KO group. The increased levels of HDL and TG content indicated that *ApoF* knockout resulted in an altered lipid profile. The above results suggest that *ApoF* knockout induces liver injury and disturbs the lipid profile.

### 3.3. Description of the m6A-Modified Genes and Peaks

**Figure 3:**
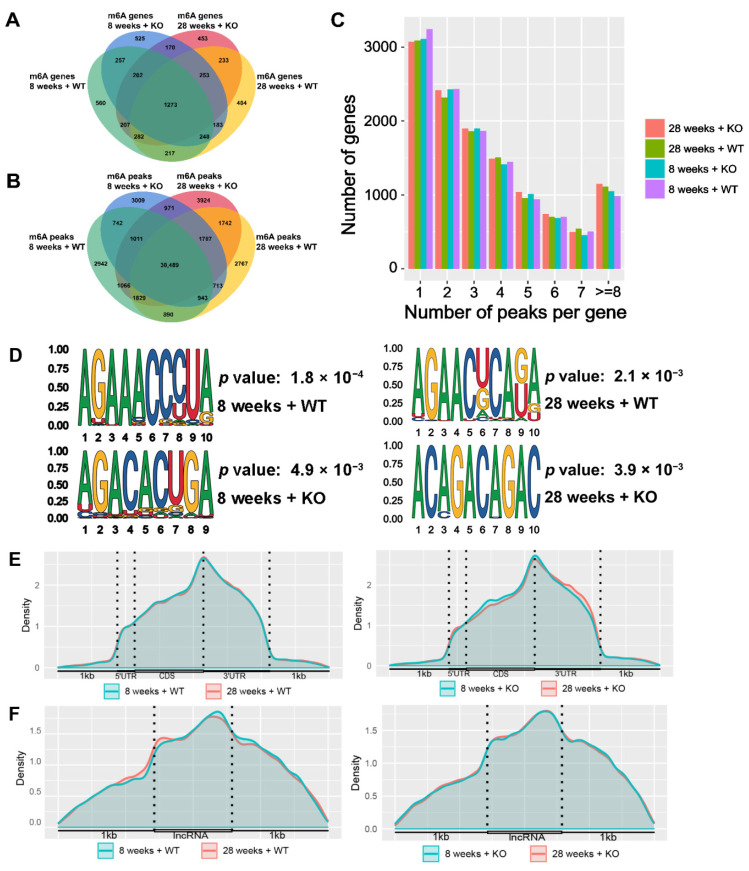
The m6A (**A**) genes and (**B**) peaks in the WT and KO mice. (**C**) The count of peaks for each gene. (**D**) The motif sequences of the m6A modification in different mice groups. Distribution of m6A peaks in (**E**) mRNAs and (**F**) ncRNAs.

As shown in Figure 3A,B, there were 3246, 3111, 3093, and 3073 m6A-modified genes, as well as 39,346, 39,665, 41,160, and 42,819 m6A peaks in the 8-week-old WT, 8-week-old KO, 28-week-old WT, and 28-week-old KO groups, respectively. A total of 1273 m6A-modified genes and 30,489 m6A peaks were found in the overlapping sets of these four groups. Regarding the number of m6A peaks in each gene (Figure 3C), most genes had one to seven peaks, and over 3000 genes had only one peak. Moreover, an excess of 1000 genes had eight or more m6A peaks.

The preferred motif sequences of the m6A peaks in the methylated mRNAs were analyzed using STREME. As shown in Figure 3D, the motif sequences were AGAAACCCUA (*p* = 0.002), AGACACUGA (*p* = 0.005), AGAACUCAGA (*p* = 0.002), and ACAGACAGAC (*p* = 0.004) in the 8-week-old WT, 8-week-old KO, 28-week-old WT, and 28-week-old KO groups, respectively. These motifs satisfied the classical RRACH sequence, where R means A or G, and H means A, C, or U, and all of them exhibited significant differences when compared to the other sequences.

As shown in Figure 3E,F, there are regularities in how m6A peaks are distributed among methylated mRNAs and ncRNAs. In the WT and KO groups, the coding sequence (CDS) and 3′-untranslated regions (3′-UTRs) contained the majority of m6A peaks, with the highest density occurring in proximity to the stop codon segment. In addition, a few m6A peaks were distributed in the 5′-UTR of the mRNAs. In the ncRNAs, the m6A peaks were evenly distributed in the sequences, and there were no significant differences between the different groups. There were also multiple m6A peaks distributed within 1 kb of the front and back of the ncRNAs.

### 3.4. DEG Annotations

**Figure 4:**
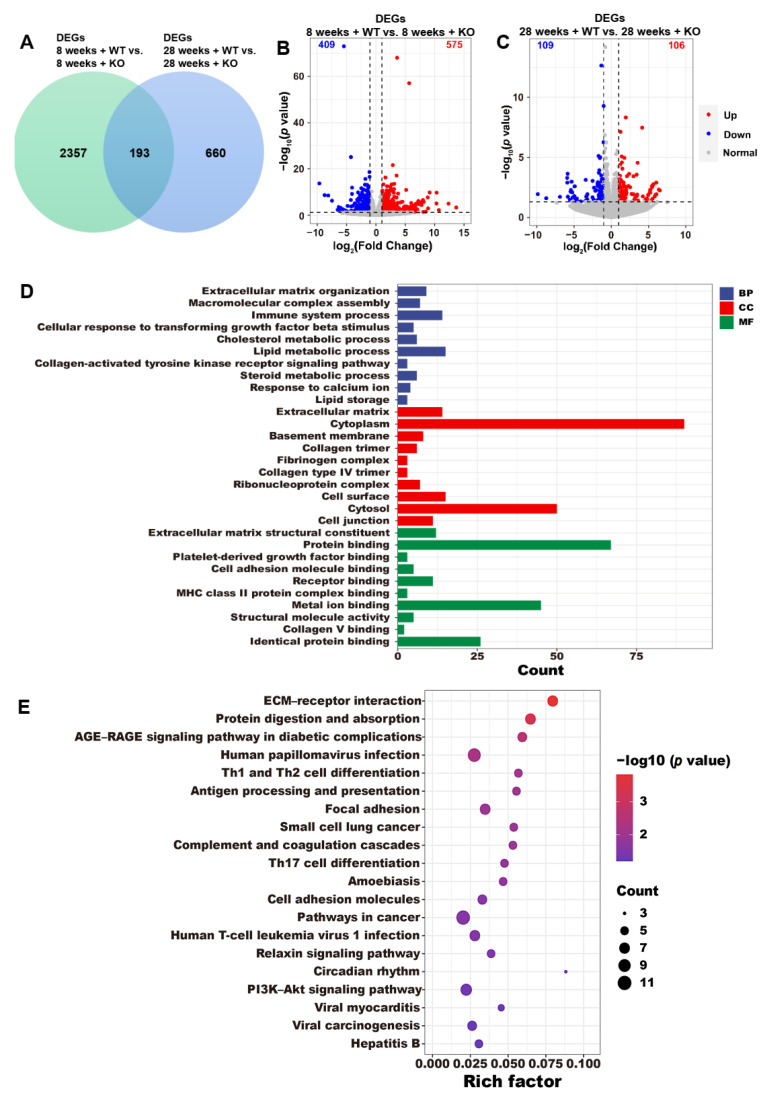
(**A**) The DEGs (*p* < 0.05) in the WT and KO mice. The volcano plots exhibit the DEGs in the WT and KO groups at different weeks with a criteria of |log_2_(Fold Change)| > 1 and *p* < 0.05 (**B**,**C**). The (**D**) GO and (**E**) KEGG enrichments of the 193 overlapping DEGs.

With a significance level of *p* < 0.05, we observed 2550 DEGs in the KO group at 8 weeks old compared to the WT group, and 853 DEGs in the KO group at 28 weeks old compared to the WT group (Figure 4A). A total of 193 genes were overlapped in these four groups. The screening standard was defined as when |log2(Fold Change)| > 1 and *p* < 0.05, 984 DEGs (575 upregulated and 409 downregulated genes) were reported between the 8-week-old KO and 8-week-old WT groups (Figure 4B), and there were 215 DEGs (106 upregulated and 109 downregulated genes) between the 28-week-old KO and 28-week-old WT groups (Figure 4C). GO (Figure 4D) and KEGG (Figure 4E) enrichment analyses were performed using the 193 overlapping genes, and the GO terms included the macromolecular complex assembly, cholesterol metabolism, lipid metabolism, steroid metabolism, response to calcium ion, and lipid storage. The KEGG terms included the ECM–receptor interaction, the AGE–RAGE signaling pathway in diabetic complications, the PI3K–Akt signaling pathway, and hepatitis B.

### 3.5. The Annotations of Differential m6A Genes

**Figure 5:**
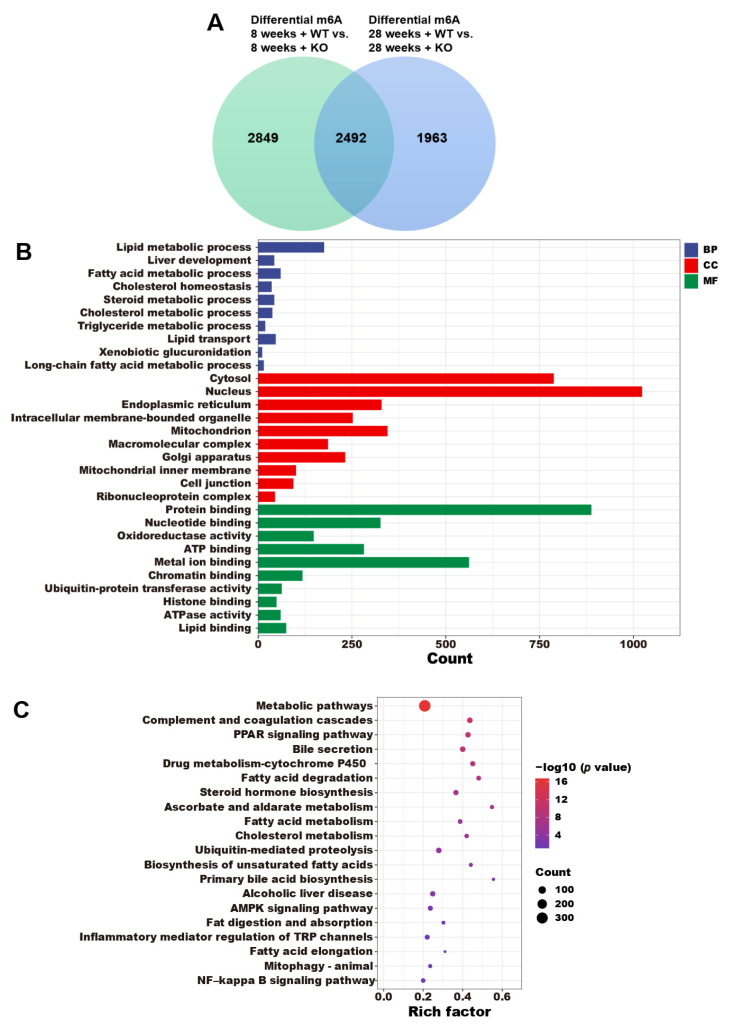
(**A**) The differentially expressed m6A genes in the WT and KO mice. The (**B**) GO and (**C**) KEGG enrichments of the 2492 overlapping differentially expressed m6A genes.

According to the data presented in Figure 5A, there were 5341 differential m6A genes between the 8-week-old KO group and the 8-week-old WT group, and 4455 differential m6A genes were identified in the 28-week-old KO group compared to the 28-week-old WT group. GO (Figure 5B) and KEGG (Figure 5C) enrichment analyses were performed by the 2492 overlapping genes, and the GO terms included lipid metabolism, liver development, fatty acid metabolism, cholesterol metabolism, TG metabolism, lipid transport, xenobiotic glucuronidation, long-chain fatty acid metabolism, and lipid binding. The KEGG terms included metabolic pathways, the PPAR signaling pathway, bile secretion, fatty acid degradation, steroid hormone biosynthesis, fatty acid metabolism, cholesterol metabolism, biosynthesis of unsaturated fatty acids, primary bile acid biosynthesis, alcoholic liver disease, fat digestion and absorption, fatty acid elongation, and the NF–kappa B signaling pathway.

### 3.6. The Annotations for Overlapping Genes between the DEGs and Differential m6A Genes

**Figure 6:**
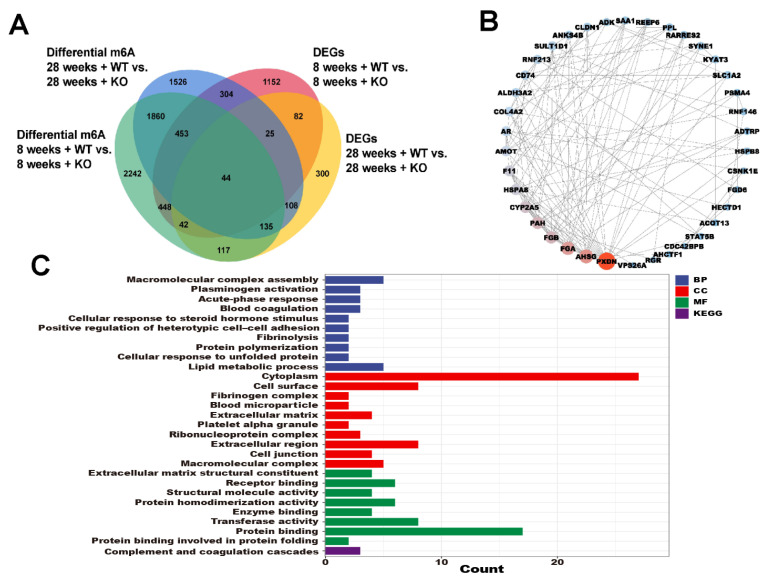
(**A**) The overlapping DEGs and differential m6A-modified mRNAs in the WT and KO mice. (**B**) The PPI of the 44 overlapping genes. (**C**) The GO and KEGG analyses of the 44 overlapping genes.

As shown in Figure 6, a total of 44 genes were found to overlap between the DEGs and differential m6A-modified mRNAs in WT and KO mice. The protein–protein interaction (PPI) network diagram illustrates the interplay among these 44 genes. The GO and KEGG terms included the macromolecular complex assembly, cellular response to steroid hormone stimulus, lipid metabolic process, extracellular matrix, and cell junction.

### 3.7. The Expression Levels of mRNA and Protein of m6A Regulators

**Figure 7:**
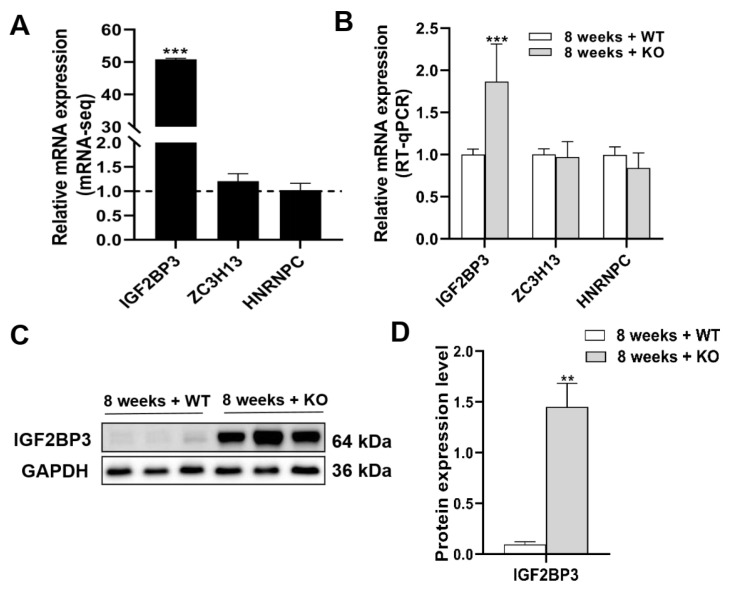
(**A**) The mRNA expression levels of IGF2BP3, ZC3H13, and HNRNPC in the 8-week-old KO group compared to the 8-week-old WT group by mRNA-seq and (**B**) RT-qPCR. (**C**,**D**) The protein expression level of IGF2BP3. *n* = 3, ** *p* < 0.01, *** *p* < 0.001.

The mRNAs acted as input, and the expression levels were detected in MeRIP-seq. Table 2 displays the mRNA expression levels of 20 m6A regulators. In the 8-week-old KO group, reader IGF2BP3 showed significant upregulation compared to the 8-week-old WT group. Similarly, in the 28-week-old KO group, reader HNRNPC was more upregulated compared to the 28-week-old WT group. RT-qPCR and Western blotting confirmed the validity of the IGF2BP3 mRNA-seq findings in the groups of mice aged 8 weeks (Figure 7). RT-qPCR showed that the mRNA expression of IGF2BP3 was approximately 50% higher in the 8-week-old KO group compared to the 8-week-old WT group (*p* < 0.001). Furthermore, in the Western blotting assay, an approximately 14-fold increase in the expression of IGF2BP3 was observed in the 8-week-old KO group as opposed to the WT group of the same age (*p* < 0.01).

### 3.8. The Role of m6A Regulators in Lipid Metabolism

**Figure 8:**
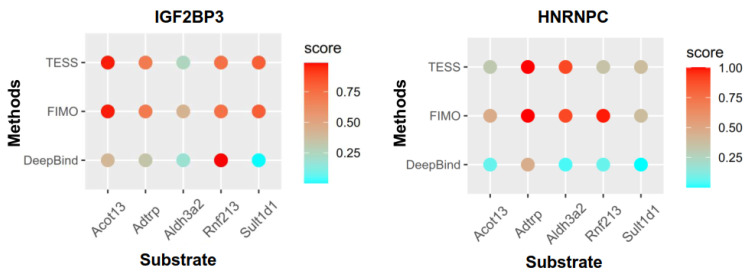
The potential substrates of the m6A regulators IGF2BP3 and HNRNPC.

A total of five counts, acyl-coenzyme a thioesterase 13 (*Acot13*), androgen-dependent tfpi-regulating protein (*Adtrp*), aldehyde dehydrogenase 3 family member a2 (*Aldh3a2*), ring finger protein 213 (*Rnf213*), and sulfotransferase family 1d member 1 (*Sult1d1*), were involved in the lipid metabolic process of the BP terms (Figure 6C). We used POSTAR3 to predict whether these mRNAs were substrates of the differentially expressed m6A regulators IGF2BP3 and HNRNPC (Figure 8). *Acot13*, *Adtrp*, *Rnf213*, and *Sult1d1* might be substrates of IGF2BP3, whereas *Adtrp* and *Aldh3a2* might be substrates of HNRNPC.

## 4. Discussion

There have been numerous reports linking lipid metabolism disorders to various diseases, which, in turn, cause serious medical and economic burdens worldwide [37,38]. Non-alcoholic fatty liver disease and hepatocellular carcinoma are classified as lipid metabolism disorder-associated liver diseases due to the excessive accumulation of lipids in hepatocytes [39,40,41]. Hepatic steatosis is positively correlated with the content of TGs. Following a 12-week period of a high-fat diet, C57BL/6J mice displayed marked increases in hepatic tissue TGs and LDLs compared to the control group, while HDL levels were decreased. The disruption of lipogenesis-related proteins, such as fatty acid synthetase, acetyl-CoA carboxylase, sterol regulatory element-binding protein-1c, and adipose differentiation-related protein, suggested abnormal lipid metabolism, which is crucial for the progression of hyperlipidemia [42]. We found serum TG was significantly increased in the *ApoF* KO groups, indicating the association between ApoF and lipid metabolism. ApoF, a 29 kDa secreted sialoglycoprotein without sequence or structural similarity to other classical Apo subtypes, mainly resides in the LDL and HDL fractions of plasma. HDL-C is considered to be a beneficial cholesterol and exerts antiatherosclerotic effects [43]. In a population study of people with normolipidemia and hyperlipidemia, the ApoF levels were 83.5 ± 4.8 μg/mL and 70.0 ± 6.3 μg/mL, and the TG contents were 93 ± 3 mg/dL and 301 ± 18 mg/dL [11]. This epidemiologic study revealed a consistent conclusion with our experimental *ApoF* KO mouse model. In our study, ALT was higher in the 28-week-old KO group compared with the 28-week-old WT group. The HDL-C levels of the KO group at the 8th week and 28th week were compared, and the difference was statistically significant (*p* < 0.01). After gene knockout of *ApoF*, the reverse cholesterol transport that transports HDL-C from the plasma to the liver was inhibited, so serum HDL-C was slightly elevated in the 28-week-old KO group compared with the 28-week-old WT group, but not significantly.

Epitranscriptomic studies have been conducted on the regulatory role of RNA chemical modifications in biological processes, which have been observed in both coding RNAs and noncoding RNAs, including mRNA, rRNA, tRNA, lncRNA, and miRNA [44,45,46]. The most common internal modification is m6A methylation, and each mRNA molecule contains around three to five methylation sites [47]. MeRIP-Seq is a high-throughput sequencing technique that uses RNA–protein immunoprecipitation to analyze m6A modification levels in RNA. These modifications affect the metabolism of target RNAs, including their maturation, splicing, export, folding, translation, and stability, thereby influencing downstream signaling pathways and physiological functions [48,49,50]. The m6A modifications primarily occur on adenines within the RRACH sequence. Regarding their distribution in the mRNA transcripts, CDS and 3’-UTR regions exhibit a significant number of m6A methylation peaks, particularly in close proximity to the stop codon region [51]. Moreover, the m6A-located regions are enriched in single nucleotide polymorphisms (SNPs), and approximately 50.2% of m6A-associated SNPs are located in the 3′-UTR. In human liver tissues, m6A-modified sites were particularly enriched in SNPs associated with lipid traits [52].

RNA m6A modifications have unique functions in regulating hepatic processes and diseases, including lipid metabolism and non-alcoholic fatty liver disease [53,54]. Lipid metabolism primarily occurs in the liver, which is responsible for lipoprotein synthesis, absorption, and transport. The regulation of m6A methylation primarily involves writers, erasers, and readers. Writers comprise a multicomponent methyltransferase complex comprising METTL3, METTL14, WTAP, VIRMA, and ZC3H13. RNA demethylases, such as FTO and ALKBH5, are erasers used for removing the m6A modification. FTO, the first identified m6A mRNA demethylase, plays a role in adipogenesis by splicing RUNX1 [55]. The m6A reader proteins, including the YTH family, IGF2BP3, and HNRNPC, could act on m6A-modified sites. RNA m6A regulators participate in lipid metabolism via multiple pathways. The enhancement of cancer proliferation, metastasis, and lipid metabolism was achieved through the regulation of stearoyl-CoA desaturase mRNA m6A modifications by the IGF2BP3-METTL14 complex [56]. The interaction between circTET2 and HNRNPC plays a crucial role in regulating the stability of carnitine palmitoyltransferase 1A, thereby impacting the lipid metabolism and proliferation of cancer cells [57]. In the writer complex, WTAP recruits METTL3 and METTL14, allowing the METTL3-METTL14 complex to influence m6A methylation [19], which could target fatty acid synthase and stearoyl-CoA desaturase 1. The current study focused on the potential regulatory mechanisms of m6A regulators in the lipid metabolic process. Our findings suggest that *Acot13*, *Adtrp*, *Rnf213*, *Aldh3a2*, or *Sult1d1* may serve as potential substrates of IGF2BP3 or HNRNPC, and A*dtrp* was identified as a common substrate based on their predictions. *Adtrp* has been identified as one of the enzymes responsible for catalyzing the hydrolysis of fatty acid esters of hydroxy fatty acids [58].

Understanding the involvement of ApoF in liver lipid metabolism is a crucial topic in the field of life and health research. This study contributes to our understanding of mRNA and m6A modification through the use of *ApoF* gene knockout mice, providing essential foundational data for future investigations. However, there are limitations to this study. While we observed differential expression of m6A readers IGF2BP3 and HNRNPC between the *ApoF* WT and KO groups, the downstream mechanism of the target genes in lipid metabolism remains unknown. Further biochemical and molecular experiments are needed to elucidate these regulatory mechanisms. Additionally, more exploration is warranted regarding the role of m6A regulators in mRNA storage and decay.

## 5. Conclusions

In the present study, transcriptome-wide MeRIP-seq and bioinformatic analyses were performed on *ApoF* knockout mice with a lipid metabolism disorder. The KO mice showed liver pathological changes, including lipid infiltration and vacuolar degeneration, as well as abnormal levels of serum TGs and HDL-C. Using high-throughput sequencing, we identified numerous DEGs and differentially m6A-modified mRNAs, which were primarily involved in lipid, fatty acid, cholesterol, and TG metabolism, as well as lipid transportation and storage. The m6A readers IGF2BP3 and HNRNPC play a crucial role in the modulation of mRNA functionality. This research offers epitranscriptomic profiles for *ApoF* KO mice and serves as a foundation for further biochemical, cellular, and in vivo studies to fully elucidate the role of ApoF in liver lipid metabolism.

## Figures and Tables

**Table 1:** The primers used for RT-qPCR.

Gene	Accession Number	Primer Sequence (5′ → 3′)	
*ApoF*	NM_133997	Forward	CACTTCACACGGAGAGGCAACC
		Reverse	GAGCAGCATCTGGCAGGACAAG
*IGF2BP3*	NM_023670	Forward	CATCTGTTTATTCCCGCCCTGTC
		Reverse	AGCCTTGAACTGAGCCTCTGG
*ZC3H13*	NM_026083	Forward	GGCAAAGAGAGTGGGAAGATAA
		Reverse	CCTGTCCTCTCGAACATGAATA
*HNRNPC*	NM_001170981	Forward	TTAATGAAAGAAATGCCCGAGC
		Reverse	CTCTGCAGCCAGGTTAATATCT
*GAPDH*	NM_001289726	Forward	ACTCCACTCACGGCAAATTCAAC
		Reverse	ACACCAGTAGACTCCACGACATAC

*ApoF*: apolipoprotein F; *IGF2BP3*: insulin like growth factor 2 mRNA-binding protein 3; *ZC3H13*: zinc finger CCCH-type containing 13; *HNRNPC:* heterogeneous nuclear ribonucleoprotein C; *GAPDH*: glyceraldehyde-3-phosphate dehydrogenase.

**Table 2:** The levels of mRNA expression for m6A regulators in the four groups.

Genes	Regulators	8-Week-Old WT vs. 8-Week-Old KO		28-Week-Old WT vs. 28-Week-Old KO	
		log_2_(Fold Change)	*p* Value	log_2_(Fold Change)	*p* Value
*IGF2BP3*	reader	5.6676	8.96 × 10^−58^	0.3463	5.66 × 10^−1^
*METTL5*	writer	0.3457	6.68 × 10^−2^	0.0420	7.95 × 10^−1^
*ZC3H13*	writer	0.2689	8.20 × 10^−2^	−0.0542	7.69 × 10^−1^
*ALKBH5*	eraser	−0.2811	1.03 × 10^−1^	−0.0004	9.98 × 10^−1^
*HNRNPA2B1*	reader	0.2657	1.15 × 10^−1^	−0.0270	8.63 × 10^−1^
*VIRMA*	writer	0.2376	1.84 × 10^−1^	−0.1127	4.81 × 10^−1^
*METTL14*	writer	0.2085	2.03 × 10^−1^	−0.0975	6.00 × 10^−1^
*IGF2BP2*	reader	0.5964	2.35 × 10^−1^	−0.4649	2.78 × 10^−1^
*FTO*	eraser	−0.1363	3.62 × 10^−1^	−0.1105	4.17 × 10^−1^
*WTAP*	writer	0.1078	4.37 × 10^−1^	0.1507	3.54 × 10^−1^
*METTL3*	writer	−0.1495	4.54 × 10^−1^	−0.0561	7.57 × 10^−1^
*YTHDF1*	reader	0.1498	4.73 × 10^−1^	−0.0304	8.99 × 10^−1^
*YTHDF2*	reader	0.1664	5.16 × 10^−1^	0.0396	8.67 × 10^−1^
*RBM15B*	writer	−0.1015	6.50 × 10^−1^	0.1865	5.26 × 10^−1^
*FMR1*	reader	−0.0769	6.66 × 10^−1^	−0.0056	9.76 × 10^−1^
*YTHDC2*	reader	−0.0711	7.36 × 10^−1^	−0.2400	2.33 × 10^−1^
*RBM15*	writer	−0.0473	7.91 × 10^−1^	0.1011	5.50 × 10^−1^
*HNRNPC*	reader	0.0240	8.64 × 10^−1^	0.4048	6.86 × 10^−4^
*CBLL1*	writer	−0.0065	9.77 × 10^−1^	0.1255	5.85 × 10^−1^
*IGF2BP1*	reader	Not detected	Not detected	−2.1825	5.87 × 10^−1^

## Data Availability

The sequence data analysis was performed by ourselves and has been uploaded to the GEO database (ID: GSE225678). It is in public mode now, and reviewers and editors can access it by visiting the following URL: https://www.ncbi.nlm.nih.gov/search/all/?term=GSE225678, accessed on 3 January 2024.
